# The Neural Basis of Fear Promotes Anger and Sadness Counteracts Anger

**DOI:** 10.1155/2018/3479059

**Published:** 2018-06-14

**Authors:** Jun Zhan, Jingyuan Ren, Pei Sun, Jin Fan, Chang Liu, Jing Luo

**Affiliations:** ^1^School of Psychology, Capital Normal University, Beijing, China; ^2^School of Marxism, Fujian Agriculture and Forestry University, Fuzhou, China; ^3^Department of Psychology, Tsinghua University, Beijing, China; ^4^Department of Psychology, The City University of New York, New York City, NY, USA; ^5^School of Psychology, Nanjing Normal University, Nanjing, China

## Abstract

In contrast to cognitive emotion regulation theories that emphasize top-down control of prefrontal-mediated regulation of emotion, in traditional Chinese philosophy and medicine, different emotions are considered to have mutual promotion and counteraction relationships. Our previous studies have provided behavioral evidence supporting the hypotheses that “fear promotes anger” and “sadness counteracts anger”; this study further investigated the corresponding neural correlates. A basic hypothesis we made is the “internal versus external orientation” assumption proposing that fear could promote anger as its external orientation associated with motivated action, whereas sadness could counteract anger as its internal or homeostatic orientation to somatic or visceral experience. A way to test this assumption is to examine the selective involvement of the posterior insula (PI) and the anterior insula (AI) in sadness and fear because the posterior-to-anterior progression theory of insular function suggests that the role of the PI is to encode primary body feeling and that of the AI is to represent the integrative feeling that incorporates the internal and external input together. The results showed increased activation in the AI, parahippocampal gyrus (PHG), posterior cingulate (PCC), and precuneus during the fear induction phase, and the activation level in these areas could positively predict subsequent aggressive behavior; meanwhile, the PI, superior temporal gyrus (STG), superior frontal gyrus (SFG), and medial prefrontal cortex (mPFC) were more significantly activated during the sadness induction phase, and the activation level in these areas could negatively predict subsequent feelings of subjective anger in a provocation situation. These results revealed a possible cognitive brain mechanism underlying “fear promotes anger” and “sadness counteracts anger.” In particular, the finding that the AI and PI selectively participated in fear and sadness emotions was consistent with our “internal versus external orientation” assumption about the different regulatory effects of fear and sadness on anger and aggressive behavior.

## 1. Introduction

**Figure 1:**
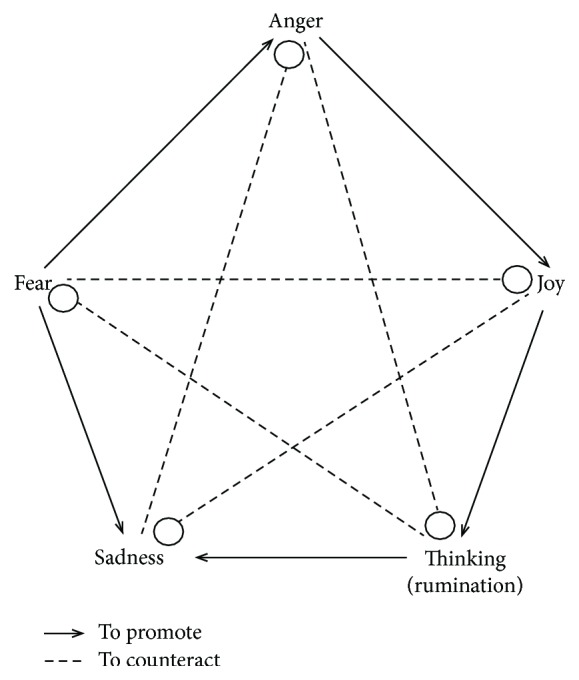
Relationships of mutual promotion and mutual restraint and the emotions of joy, thinking/anxiety (The original word for “thinking” in the Chinese literature is 思 [read as si]; 思 may indicate either the pure cognitive thinking and reasoning process that is nonpathogenic or the maladaptive repetitive thinking or ruminative thinking that is typically associated with negative emotion and has pathogenic potential. Thus, 思 may have different meanings in different contexts of the MPMC theory. The implication of maladaptive “thinking” in the MPMC theory of emotionality includes not only ruminative thought per se but also the negative, depression-like emotion associated with it. Therefore, in specific contexts, particularly the context discussed in this study, 思 indicates the ruminative or repetitive thinking that is closely related to rumination in modern psychology, which is defined as a pattern of repetitive self-focus and recursive thinking focused on negative cases or problems (e.g., unfulfilled goals or unemployment) that is always associated with the aggravation of negative mood states (e.g., sadness, tension, and self-focus) and has been shown to increase one's vulnerability to developing or exacerbating depression [4].), sadness, fear, and anger. The promotion relationships include the following: joy promotes thinking/anxiety, thinking/anxiety promotes sadness, sadness promotes fear, fear promotes anger, and anger promotes joy. The restraint relationships include the following: joy counteracts sadness, sadness counteracts anger, anger counteracts thinking/anxiety, thinking/anxiety counteracts fear, and fear counteracts joy.

Western psychology generally advocates the use of cognitive methods, such as rational or cognitive reappraisal, to downregulate negative emotions. However, hormones released in response to stress can impair the advanced function of the prefrontal cortex (PFC), leading to a failure of cognitive reappraisal in regulating conditioned fear under stress [1, 2]; thus, emotion regulation strategies that are less reliant on the PFC could be more suitable for changing negative responses to emotional arousal under stress than normal downregulating strategies [3]. In contrast to cognitive emotion regulation theories, traditional Chinese philosophy and medicine consider different types of emotions to have mutual promotion and mutual counteraction (MPMC) relationships (Figure 1) involving a down-up process that depends less on the PFC [4], thereby suggesting a novel approach for emotion regulation that may overcome the shortcomings of traditional cognitive regulation strategies.

In our recent study, aggressive behavior associated with anger was found to be effectively reduced by inducing sadness, while the induction of fear significantly increased self-reported anger; these findings provided behavioral evidence supporting the hypotheses proposed by the MPMC theory of emotionality that suggest “sadness counteracts anger” and “fear promotes anger” [3, 4]. In that experiment, anger was first induced by asking the participants to read an extremely negative comment regarding their viewpoints (the mutual article evaluation paradigm) or watch standardized anger-inducing movie clips; then, fear, sadness, or a neutral mood was induced. The participants who were provoked exhibited less aggressive behavior if sadness was subsequently induced; however, the participants became increasingly angry if fear was subsequently induced.

More importantly, the principle of “sadness counteracts anger” may have application value because the induction of sadness (e.g., passively watching a clip from a sad movie or listening to sad music) requires obviously fewer cognitive control resources mediated by the PFC and may regulate negative emotion; therefore, this principle could have some advantages in regulating emotion relative to cognitive-regulation strategies that may fail to work under stress. To test this hypothesis, in our recent study, we directly compared the effects of cognitive reappraisal and sadness induction on reducing anger or anger-related aggression in nonstressful and stressful situations [3]. Expectedly, cognitive reappraisal was unable to effectively relieve the subjective feeling of anger under the stress condition; however, the stressful condition did not influence the efficiency of sadness induction in reducing aggressive behavior. First, all the participants were assigned to a nonstressful or stressful condition and were provoked using the mutual article evaluation paradigm; then, the participants were asked to make a cognitive reappraisal or watch sad movie clips. The cognitive reappraisal effectively reduced self-reported anger under the nonstress condition but failed to have such an effect under the stress condition; meanwhile, high cortisol levels were found to be maintained in and after the reappraisal. It is possible that cortisol activation triggered by the arousal of the hypothalamic-pituitary-adrenal (HPA) axis disrupted the PFC function and further impaired the efficiency of cognitive regulation, while stress did not influence the effects of sadness induction on aggressive behavior and related skin conductance, suggesting that the emotion regulation strategy is relatively immune to stress.

However, the cognitive brain processes underlying the phenomenon of “sadness counteracts anger” and “fear promotes anger” are still unknown. A general perspective for understanding these mechanisms is to consider the ways in which different types of emotions interact, that is, how an antecedent or subsequent emotion (such as sadness or fear) could interact with the targeted emotion (such as anger). This investigation of the process and neural mechanism of the interactions among different emotions could increase our understanding of the effective principle of the “sadness counteracts anger” strategy. For example, if an individual is aroused by sadness or fear before or after being provoked, a certain pattern of neuropsychological components activated by the sadness or fear could affect the expression of anger or aggressive behavior.

More specifically, according to a meta-analysis of the neural activation patterns associated with different types of basic emotions, the anger and fear categories both prioritized cortical processes that support an “external orientation/object-focused” schema, which is characterized by goal-driven responses in which objects and events in the world are in the foreground [5]. In contrast, the cortical patterns associated with sadness support an internal orientation/homeostatic-focused schema characterized by an orientation toward immediate somatic or visceral experiences, which prioritizes the processing of interoceptive and homeostatic events [5]. Thus, the neural circuits mediating anger and related aggression may be more easily triggered by the neural activity underlying fear but more efficiently eliminated by the neural activity underlying sadness [4].

To test this hypothesis, this study investigated the regulatory effects of antecedently induced sadness or fear on the subsequent anger and related aggressive behavior in a provoking situation and analyzed the accompanying brain mechanisms using functional magnetic resonance imaging (fMRI). Specifically, we explored and verified the possibility that following antecedent-induced sadness, individuals are less likely to become angry or aggressive (“sadness counteracts anger”) in a provoking situation, while following antecedent-induced fear, individuals are more likely to become angry or display more aggression (“fear promotes anger”) in a provoking situation. We identified the key brain regions activated by sadness or fear inducing and further analyzed the correlation between the activations of these regions and the subsequent anger-related responses in subsequent provocation.

In particular, we made an “internal versus external orientation” assumption proposing that fear could promote anger because of its external orientation associated with motivated action, whereas sadness could counteract anger because of its internal or homeostatic orientation to somatic or visceral experience. This assumption could be examined by detecting the selective involvement of the posterior insula (PI) and the anterior insula (AI) in sadness and fear. According to the theory of the posterior-to-anterior progression of insular function in re-representing human feeling and emotion, the PI represents more primary quantities, whereas the AI integrates more contextual information in its representation of emotion [6, 7]. Therefore, we propose that fear could be more intensively represented in the AI by its external encoding or contextual integrating orientation and that this orientation, because of its similarities with anger, will promote anger-related feeling and behavior, whereas sadness could be more intensively represented in the PI by its internal orientation or homeostatic-focused schema and that this orientation, because of its dissimilarities with anger, will counteract with anger-related feeling and behavior.

## 2. Materials and Methods

### 2.1. Participants

The sample size was 24, which was calculated with the G^∗^Power software 3.1.9.2 (input parameter: *α*: 0.05; power (1 − *β*): 0.8). In addition, to minimize the potential impact of age differences, twenty-six college students (17 females and 9 males, aged 19–25 years, mean *age* = 22 years, all native Chinese speakers) at universities in Beijing were recruited to participate in this study as paid volunteers. All the participants were right-handed, had normal or corrected-to-normal vision, and had no history of neurological or psychiatric problems. Prior to the scanning session, the participants signed informed consent forms, and the study was approved by the Institutional Review Board of the Center for Biomedical Imaging Research of Tsinghua University. After the experiment, each participant was compensated with 120 RMB for participating in the study. Two participants (1 male and 1 female) were excluded from the analysis due to excessive head motion during the scanning.

### 2.2. Experimental Design and Procedures

#### 2.2.1. Overview of the Experimental Procedure

In contrast to the experimental procedure used in our previous study, which examined the regulatory effects of subsequently evoked sadness or fear on the anger emotion that had already been evoked [3, 4], in this study, we adopted a modified experimental procedure to examine the interaction between anger and sadness or fear, which could be more suitable for within-subject design. We examined the inhibitory or facilitatory effects of the antecedently evoked sadness or fear on anger or aggressive behavior in an offensive situation subsequently experienced by the participants. A single-factor (mood induction: fear versus sadness versus neutral mood) within-subject design was adopted in this study in which the participants experienced three episodes of fear, sadness, or neutral emotion induction, and each emotion induction was followed by a modified competitive reaction time task to provoke the participants; the level of subjective anger was measured at baseline and after the competitive reaction time task. Using this paradigm, a within-subject design that is more suitable for an fMRI investigation could be applied.

#### 2.2.2. Evaluation of Subjective Anger

The subjective feeling of anger was measured using the hostility subscale of the revised Multiple Affect Adjective Checklist (MAACL) [8, 9]. In the Chinese version of the MAACL [10], the hostility subscale contains 22 adjectives, including 11 words that are positively associated with anger (i.e., irritable, cruel, jealous, disgruntled, indignant, impatient, hostile, irritated, violent, furious, and exasperated) and 11 words that are negatively associated with anger (i.e., gracious, easy-going, good-natured, helpful, friendly, courteous, gentle, pleasantly agreeable, kind, affable, and cooperative). All the participants were required to assess these 22 adjectives according to their current feelings and to select each positive anger word (press the “1” button) or to unselect each negative anger word (press the “2” button). Each selection accumulated one point, and the final scores were the sum of the total points of the selected positive anger words and unselected negative anger words. A high total score indicated a high level of anger.

#### 2.2.3. Fear/Sadness/Neutral Mood Induction

In this study, 3 video clips were used to induce fear (duration, 2 min 20 sec; from the movie “Help”; intensity, M = 3.33, SD = 2.1), sadness (duration, 2 min 20 sec; from the movie “Mom Love Me Once Again”; intensity, M = 3.17, SD = 1.56), and a neutral emotion (duration, 2 min 20 sec; from the movie “Computer Repair”; intensity, M = 1.0625, SD = 0.25). The movie clips were extracted from the Chinese Emotional Visual Stimulus (CEVS) database [11]. While watching the clips, the participants were asked to be as attentive to the clips as possible, to express their natural feelings, and to avoid suppressing any emotion.

#### 2.2.4. Anger Induction and Aggressive Behavior Measure

The Taylor Aggression Paradigm (TAP) was used to induce anger and measure aggressive behaviors [12–15]. The modified version of the TAP was used in this study, and the task paradigm was adopted from a previous study [16]. In this task, the participants were informed that they would be playing 24 successive competitive reaction-time trials against an opponent. However, there was no opponent, and the entire program was established in advance. At the beginning of each trial, the participant was shown the opponent number for the upcoming competition (each of the three runs was supposedly played against a different opponent to avoid the possible influence of the competition experience with the opponent against whom they had competed in the previous run). Each participant was allowed to determine the intensity of the noise, that is, between 65 decibels (1—very weak) and 95 decibels (4—very strong), his/her opponent would hear if the opponent lost; each noise had a 2-second duration. Participants were instructed to select the noise intensity from 1 to 4 before each competition trial, and the average noise intensity selected by the participants over 24 rounds was used to indicate participants' aggression levels. After the participants selected the noise intensity, they were provoked by being shown the high-punishment selection (level 3 or 4, each 50%) of their opponents. Finally, feedback was provided regarding whether the participant won or lost. In the losing trials, the participants were exposed to aversive noise; in the winning trials, the participants did not receive a punishment. However, all win and fail trials were secretly controlled by the experimenter, and the participants won 12 of the 24 trials of the competition game.

### 2.3. Imaging Procedure

**Figure 2:**
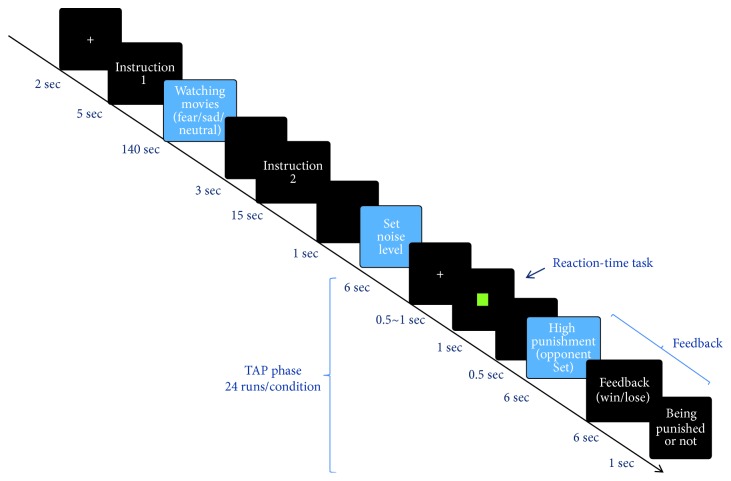
Overview of the presentations experienced by each subject over the course of the experiment. The entire experiment consisted of three runs (i.e., fear/sadness/neutral mood conditions) of the procedure.

The scanning was divided into three runs according to the mood induction (fear, sadness, or neutral mood), and the run sequence was balanced across all participants. The interval between two runs was 3 min to allow the participants' mood to return to the baseline level and minimize any carryover effect [17]. The duration of each run was 12 min and 54 sec, and the total time of the functional imaging was 38 min and 42 sec. Each run consisted of two sessions (Figure 2). The first session included the phases of “introduction 1” and “watching movies.” During “introduction 1,” the participant was required to pay attention to watching movies, and in the phase of “watching movies,” the participant was assigned to watch one of three different emotional movie clips to induce sadness, fear, or neutral emotions, with a clip duration of 140 sec. During the second session, “instruction 2” was used to introduce the rules of the TAP, which consisted of 24 trials and was used to elicit and assess aggression [16]. Each trial included the phase of “set noise level” (duration: 6 sec), in which the participant set the noise intensity for the opponent; the phase of “reaction-time task” (duration: 1 sec), in which the participant played against an opponent; and the phase of “feedback” (duration: 13 sec), in which the participant was provoked by being shown the opponent's high-punishment selection.

### 2.4. Image Acquisition

The data were acquired from the Center for Biomedical Imaging Research of Tsinghua University. The fMRI scanning was performed using a 3 T magnetic resonance scanner (Philips, Netherlands) with a 32-channel frequency head coil. To restrict head movements, the participants' heads were fixed with plastic braces and foam pads during the entire experiment. To perform the functional imaging, we used an echo-planar sequence based on blood oxygenation level-dependent (BOLD) contrast with the following parameters: time (TR) = 2000 ms, echo time (TE) = 35 ms, flip angle (FA) = 90°, field of view (FOV) = 200 mm × 200 mm, 64 × 64 matrix, voxel size = 2.5 × 2.5 × 4 mm^3^, 30 slices, and 4 mm thickness. T2^∗^-weighted function images parallel to the anterior commissure-posterior commissure (AC-PC) were obtained. To obtain structural images, high-resolution structural T1^∗^-weighted anatomical scanning was performed using a 3D gradient-echo pulse sequence (TR = 7.65, TE = 3.73, flip = 90°, FOV = 230 mm × 230 mm, and voxel size = 0.96 mm × 0.96 mm × 1 mm).

### 2.5. Image Analysis

The imaging data were analyzed using SPM 8 (Statistical Parametric Mapping, Wellcome Department of Cognitive Neurology, London, UK). During preprocessing, the images of each participant were corrected with slice-timing, realigned to correct for head motion, spatially normalized into a standard echo planar imaging (EPI) template in the Montreal Neurological Institute (MNI) space, and smoothed using an 8 mm Gaussian kernel full width at half maximum (FWHM).

For each participant, a general linear model with eighteen events was defined. Specifically, each run consisted of six events, including “instruction 1,” “watching movies,” “instruction 2,” “set noise level,” “reaction-time task,” and “feedback” (merged with “the presentation of a high punishment by the opponent,” “feedback regarding winning or losing,” and “being punished or not punished”). Because each run included one experimental condition (fearful, sad, or neutral), the three runs had eighteen (3 runs × 6 events/run) events (Figure 2 and Table 1). All the events were modeled with a canonical hemodynamic response function using the standard SPM8 settings. Six covariates (i.e., three rigid-body translations and three rotations resulting from the realignment) were also included to account for movement-related variability. Regionally specific condition effects were tested with performing linear contrasts for each key event relative to the baseline and each participant.

During the mood-induction phase, we were primarily interested in the differences in the cognitive brain responses among the different mood inductions (i.e., fear versus neutral mood induction, fear versus sadness induction, sadness versus neutral mood induction, and sadness versus fear induction). We additionally performed conjunction analyses of “fear induction > neutral mood induction,” “fear induction > sadness induction,” “sadness induction > neutral mood induction,” and “sadness induction > fear induction” to identify the selective effects of the fear and sadness inductions.

The threshold of the whole-brain analyses was generally set at the threshold of *p* < 0.001 (uncorrected for multiple comparisons). All ROIs were created by superimposing the activated clusters obtained from the given contrast (e.g., the parahippocampal activation obtained in the conjunction analysis of “fear induction > neutral mood induction” and “fear induction > sadness induction”) on the mask defined in the WFU PickAtlas (Version 3.0, http://fmri.wfubmc.edu/software/PickAtlas), and the percentage signal changes were extracted from MarsBar (http://marsbar.sourceforge.net). The percentage signal changes within each ROI were extracted separately for each participant under each condition.

## 3. Results

### 3.1. Behavioral Results

**Figure 3:**
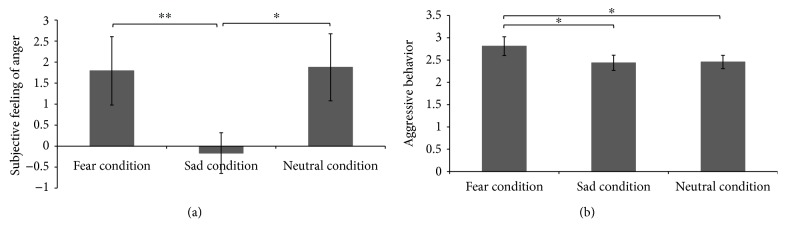
Comparison of the subjective feeling of anger and aggressive behavior under the fear, sad, and neutral conditions. The difference between the subjective anger feeling in each condition and that at baseline is shown in (a). The aggressive behavior under the three conditions is shown in (b). The error bars (capped vertical bars) represent (−1)/(+1)SE. ^∗∗^ indicates a significant difference at *p* < 0.01; ^∗^ indicates a significant difference at *p* < 0.05.

The change in subjective anger (the difference between subjective anger after the TAP session and at baseline) was significantly lower under the sadness condition than under the fear [*t*(23) = −2.964, *p* < 0.05, *d* = 0.526] and neutral mood [*t*(23) = −2.553, *p* < 0.05, *d* = 0.470] conditions, and no significant differences were observed between the fear and neutral mood conditions (Figure 3(a)). In addition, aggressive behavior, as determined with the average noise intensity set by the participant to punish his/her opponent over 24 rounds of the competition, under the fear condition was significantly higher than under the sadness [*t*(23) = 2.382, *p* < 0.05, *d* = 0.445] and neutral mood [*t*(23) = 2.384, *p* < 0.05, *d* = 0.445] conditions, and no significant differences were observed between the sadness and neutral mood conditions (Figure 3(b)).

### 3.2. Imaging Results

**Figure 4:**
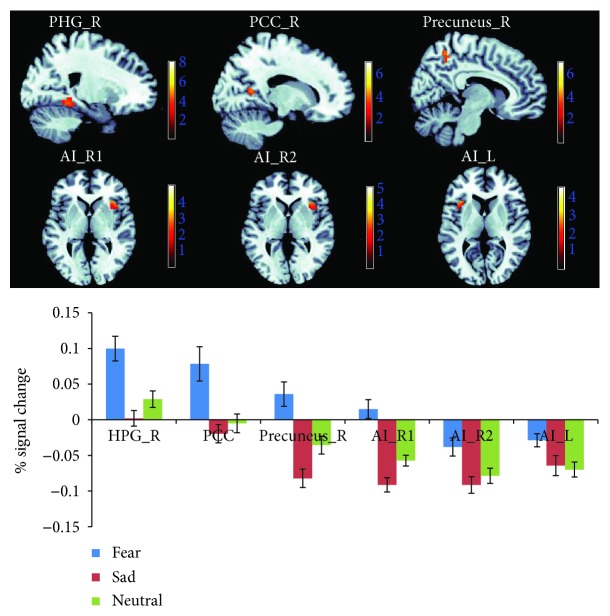
Neuroimaging results showing brain activation associated with fear induction (i.e., watching movies). The activation of the PHG_R, PCC_R, and precuneus_R result was taken from the conjunction analysis of “fear > neutral” and “fear > sadness” (depicted at threshold of *p* < 0.001), the activation of AI_R1 was taken from the contrast of “fear > sadness” (depicted at threshold of *p* < 0.05), and the activation of AI_R2 and AI_L was taken from the contrast of “fear > neutral” (depicted at *p* < 0.05). The graphs show the mean percent signal changes for the PHG_R, PCC_R, precuneus_R, AI_R1, AI_R2, and AI_L across the three experimental conditions.

**Figure 5:**
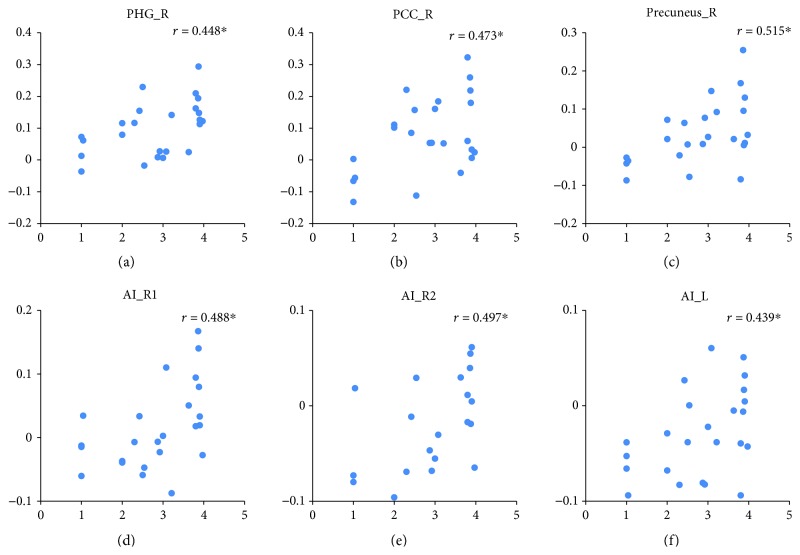
Relationships between brain activation associated with fear induction and aggressive behavior under the fear condition. *r* represents the correlation coefficient. ^∗^ indicates a significant difference at *p* < 0.05.

The effect of fear induction was examined using the contrasts of “fear induction > sadness induction” and “fear induction > neutral mood induction” (both sampled during the emotional movie-clip-viewing) and with the conjunction analyses of these two contrasts. Increased neural activity selectively associated with fear was identified in the right parahippocampal gyrus (PHG_R, BA19), right posterior cingulate cortex (PCC_R, BA30), and right precuneus (precuneus_R, BA7) by the conjunction analyses of “fear induction > sadness induction” and “fear induction > neutral mood induction.” Right anterior insula (AI_R, BA13) and left anterior insula (AI_L, BA13) activation was detected in both contrasts of “fear induction > sadness induction” and “fear induction > neutral mood induction” but was located in different AI regions in these two contrasts. Thus, the conjunction analysis did not identify AI activation (Figure 4 and Table 2). In addition, under the fear condition, the BOLD responses in the ROIs of the PHG, PCC, precuneus, and AI positively predicted the subsequent aggressive behavior levels (i.e., noise intensity determined by the participants to punish their opponents) [*r*_PHG_R_ = 0.488, *p* < 0.05; *r*_PCC_R_ = 0.473, *p* < 0.05; *r*_precuneus_R_ = 0.515, *p* < 0.05; *r*_AI_R(fear > sadness)_ = 0.488, *p* < 0.05; *r*_AI_R (fear > neutral)_ = 0.497, *p* < 0.05; and *r*_AI_L (fear > neutral)_ = 0.439, *p* < 0.05] (Figure 5).

**Figure 6:**
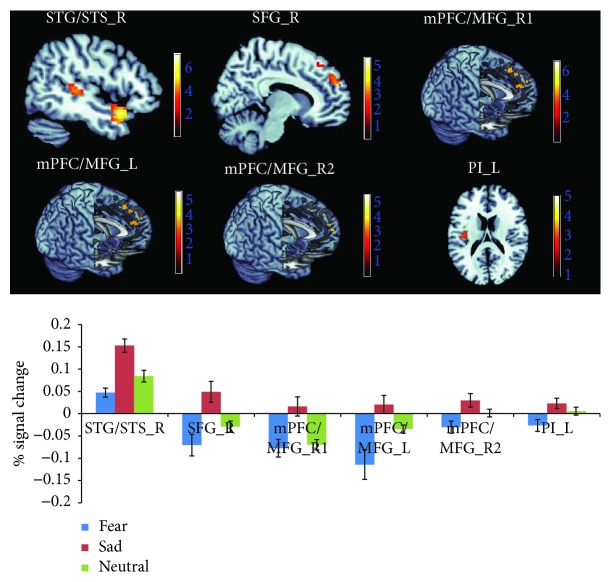
Neuroimaging results showing brain activation associated with sadness induction (i.e., watching movies). The activation of the STG/STS_R and SFG_R was taken from the conjunction analysis of “sadness > neutral” and “sadness > fear” (depicted at *p* < 0.001), the activation of the mPFC/MFG_R1 and mPFC/MFG_L was taken from the contrast of “sadness > fear”, the activation of the mPFC/MFG_R2 was taken from the contrast of “sadness > neutral” (depicted at *p* < 0.05), and the activation of PI was taken from the contrast of “sadness > fear” (depicted at *p* < 0.005). The graphs show the mean percent signal changes separately for the STG/STS_R, SFG_R, mPFC/MFG_L, mPFC/MFG_R2, and PI_L across the three experimental conditions.

**Figure 7:**
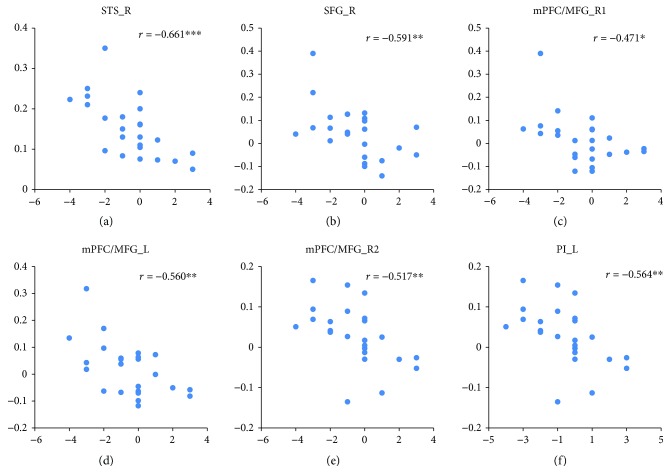
Relationships between brain activation associated with sadness induction and subjective anger feelings under the sadness condition. *r* represents the correlation coefficient. ^∗∗∗^ indicates a significant difference at *p* < 0.001, ^∗∗^ indicates a significant difference at *p* < 0.01, ^∗^ indicates a significant difference at *p* < 0.05.

The effects of the sadness induction were examined by the contrasts of “sadness induction > fear induction” and “sadness induction > neutral mood induction” (both sampled during the emotional movie-clip-viewing) and by the conjunction analyses of these two contrasts. Increased neural activity selectively associated with sadness induction was identified in the right superior temporal gyrus/sulcus (STG/STS_R, BA 22/38/41) and right superior frontal gyrus (SFG_R, BA9) by the conjunction analysis. Left and right medial prefrontal cortex/medial frontal gyrus (mPFC/MFG_L, mPFC/MFG_R) activation was detected in both contrasts of “sadness induction > fear induction” and “sadness induction > neutral mood induction,” but the exact location in the mPFC/MFG differed between these two contrasts (Figure 6 and Table 3), and left posterior insula (PI_L) activation was only detected in the contrast of “sadness induction > fear induction.” Under the sadness condition, the BOLD responses in the ROIs of the STG/STS, SFG, mPFC/MFG, and PI were negatively correlated with the subjective anger feeling [*r*_STG/STS_R_ = −0.661, *p* < 0.001; *r*_SFG_R_ = −0.519, *p* < 0.01; *r*_mPFC/MFG_R (sadness > fear)_ = −0.471, *p* < 0.05; *r*_mPFC/MFG_L (sadness > fear)_ = −0.560, *p* < 0.01; *r*_mPFC/MFG_R (sadness > neutral)_ = −0.517, *p* < 0.01; and *r*_PI_L(sadness > fear)_ = −0.564, *p* < 0.01] (Figure 7).

## 4. Discussion

In the current study, the participants showed more aggressive behavior after they were induced with fear and a lower level of anger after they were induced with sadness, thus supporting the hypotheses of the MPMC theory of emotionality that “sadness counteracts anger” and “fear promotes anger” from a “proactive interference perspective” that is different from the “retroactive interference perspective” in our previous studies [3, 4]. In our previous study, participants were first provoked, and we found that afterward-induced sadness could reduce the subsequent aggressiveness level, whereas afterward-induced fear promoted angry feelings [3, 4]. Therefore, the MPMC theory principle of “sadness counteracts anger” may refer to the following two different situations: the subsequently induced sadness could help to control anger-related aggressive behavior (the retroactive regulatory effects) and the antecedently induced sadness could help to reduce angry feelings (the proactive regulatory effects). Similarly, the principle of “fear promotes anger” also involves the following two situations: feelings of anger could increase if fear is subsequently experienced, indicating that fear promotes existing anger (the retroactive regulatory effects), and an individual may express more aggressive behavior during an aggravating situation if he/she is antecedently evoked by fear, indicating that existing fear could foster aggressive behavior (the proactive regulatory effects). Interestingly, the principles of “sadness counteracts anger” and “fear promotes anger” have different effects on subjectively reported anger and aggressive behavior in their retroactive or proactive regulation form. In the retroactive regulation form, “sadness counteracts anger” significantly reduces aggressive behavior, whereas in the proactive regulation form, “sadness counteracts anger” significantly reduces anger. Similarly, in the retroactive regulation form, “fear promotes anger” significantly promotes anger, whereas in the proactive regulation form, “fear promotes anger” significantly promotes aggressive behavior. Thus, aggressive behavior, despite its close relationship with anger [12, 18], may be selectively regulated in different ways depending on the context. Further studies should investigate the difference between anger and aggressive behavior in terms of their regulatory approaches and context.

The main goal of this study was to explore the cognitive brain mechanism underlying the principles of “fear promotes anger” and “sadness counteracts anger.” Compared with the sadness and neutral mood induction, the fear mood induction was associated with more activation in the AI, PHG, PCC, and precuneus, and activation in these regions could positively predict the individuals' anger feelings in a subsequent provocation situation. However, compared with the fear and neutral mood inductions, the sadness mood induction was associated with more activation in the PI, STG/STS, and SFG, and the activation in these regions could negatively predict the individuals' aggressive behavior in subsequent provocation situations.

First, the AI, PHG, PCC, and precuneus activation was associated with the processing of the fear-inducing movie clip, which is consistent with previous neuroscience studies showing that fearful or threatening stimuli elicit activity in the AI, hippocampus, PCC, and precuneus, and this activation is mainly characterized by wakefulness and goal-driven responses [5, 19]. Second, areas in the PI, frontal lobe (e.g., superior frontal gyrus and medial frontal gyrus) and superior temporal gyrus were selectively activated during the processing of the sadness-inducing movie clip, which is also consistent with previous studies investigating the neural correlates of sadness [20–23]. These findings, together with the significant correlation between the brain activation and subsequent anger or aggressive behavior, may imply the possible neural mechanism of “fear promotes anger” and “sadness counteracts anger.”

Most importantly, our results demonstrate a clear functional dissociation between the AI and PI in which the AI is more involved in fear induction, and this AI activation positively predicted later anger, whereas the PI was more involved in sadness induction, and this PI activation negatively predicted later aggressive behavior. This result not only proved that sadness and fear could be different in their representation location in the posterior-to-anterior progression of insular structure but also implied that the mechanism mediating the different inducing effects of sadness and fear on anger and aggressive behavior could be related to this difference. The AI is generally considered a part of the neural loop that notices, evaluates, and adapts to threat signals [7, 24]; the AI also reflects negative emotions, such as anxiety, aversion and alertness, arising from individual conflicts in the face of unfair events [25]. The AI activation in fear, together with the activation in the PHG, PCC, and precuneus, which could be related to conscious information processing such as attentive focusing and awakening [26–29], implied that the reason fear enhanced aggressive behavior could be attributed to an externally oriented threat-driven arousal state. Different from fear, sadness tended to be selectively represented in the PI and was associated with the representation of feeling oriented toward one's internal feeling and experience. The PI has been shown to connect reciprocally with the secondary somatosensory cortex and is highly specialized to convey homeostatic information such as pain, temperature, itch, and sensual touch [6, 30, 31], and a number of studies indicate that a subsection of the PI both anatomically and functionally serves a primary and fundamental role in pain processing [30, 32–34]. In addition, the induction of sadness was also accompanied by the empathy- or sympathy-related neural processing process embodied by the activation of STG/STS and mPFC [35, 36]. Previous studies suggested that STS was engaged in tasks that required one to infer and share in another individual's mental [37, 38] and emotional state [39, 40]. For example, Zelinková and colleagues found that videos depicting dangerous behavior in a traffic campaign ending with tragic consequences activated the STS and that this activation was directly related to the participants' empathy and sympathy [41]. Thus, the possible neurological basis of “sadness counteracts anger” is that sadness induced internally oriented feeling represented in the PI, while eliciting empathy and sympathy processes mediated by STS/STG and mPFC, and finally producing less of a tendency to feel anger when provoked by others.

The current findings and conclusions must be considered in light of our study's limitations. First, as discussed above, the self-reported anger and aggressive behavior were inconsistent because the sadness induction successfully decreased the self-reported anger but not aggressive behavior; thus, whether the target of “fear promotes anger” or “sadness counteracts anger” occurs at the cognition or behavior level or both requires further confirmation in future studies. Second, we only examined 24 healthy, young Chinese college students. Thus, our findings cannot be generalized to larger populations, a nationality-unspecific context, or any clinical population. Finally, the mood (fear, sadness, or anger) inductions in this study were almost controlled in a moderate intensity. Regulating different intensities of emotional stimuli, however, may involve different neural mechanisms [42]; thus, studies should investigate the influence of the inducing mood intensity on the neural responses of “fear promotes anger” and “sadness counteracts anger.”

## 5. Conclusions

In summary, our findings suggest a clear functional dissociation between the anterior and posterior parts of insula in which the AI is more involved in the processing of “fear promotes anger” than the PI and the PI is more involved in the processing of “sadness counteracts anger” than the AI. Specifically, fear-induced AI activity is associated with negative feelings (e.g., disgust and cognitive conflict) and neural responses are related to arousal (PHG, PCC, and precuneus), further promoting more aggression to external irritation. In contrast, sadness elicited the activation of the PI, which is involved in the processing of primary feeling and neural regions that may be related to empathy/sympathy (STG/STS, SFG, and mPFC), further producing less of a tendency to feel anger when provoked by others. These findings provide compelling neurological evidence supporting the “fear promotes anger” and “sadness counteracts anger” hypotheses of the MPMC theory of emotionality, which is based on traditional Chinese medicine.

## Figures and Tables

**Table 1:** Illustration of the eighteen events defined in the image analysis.

Number	Run—condition	Session	Event	Onset time (sec)	Duration (sec)
1	Run 1—fear	Fear induction	Instruction 1	20	5
2			Watching fear movie clip	25	140
3			Instruction 2	168	15
4		TAP (first trial)	Determining noise level	186	6
5			Reaction time task	192	1
6			Provocation	193	13

7	Run 2—sadness	Sadness induction	Instruction 1	20	5
8			Watching sad movie clip	25	140
9			Instruction 2	168	15
10		TAP (first trial)	Determining noise level	186	6
11			Reaction time task	192	1
12			Provocation	193	13

13	Run 3—neutral	Neutral induction	Instruction 1	20	5
14			Watching neutral movie clip	25	140
15			Instruction 2	168	15
16		TAP (first trial)	Determining noise level	186	6
17			Reaction time task	192	1
18			Provocation	193	13

**Table 2:** Brain regions associated with the effects of fear induction.

Brain regions	Hemisphere	Brodmann's area	MNI coordinates			*t*(24)	*k*
			*x*	*y*	*z*		
*(fear > neutral) ∩ (fear > sadness) (conjunction)*							
Parahippocampal gyrus	Right	19	24	−46	−5	4.46	48
Culmen	Left		−18	−46	−8	4.42	58
Posterior cingulate	Right	30	18	−55	13	4.02	19
Precuneus	Right	7	9	−49	55	3.96	27
Precuneus	Right	7	9	−52	46	3.37	
Cingulate gyrus	Left	31	−15	−37	43	3.82	7
Uvula	Left		0	−70	−29	3.77	6
Claustrum	Right		30	29	1	3.69	9
*fear > sadness*							
Parahippocampal gyrus	Right	36	30	−46	−11	7.35	214
Parahippocampal gyrus	Left	36	−30	−40	−11	7.14	290
Fusiform gyrus	Left	37	−30	−52	−11	6.95	
Declive	Left		−30	−67	−14	3.33	
Cingulate gyrus	Left	31	−15	−37	43	6.8	1270
Middle occipital gyrus	Right	19	42	−79	19	5.8	
Precuneus	Right	7	9	−52	55	5.78	
Middle temporal gyrus	Left	19	−36	−82	28	5.06	89
Middle occipital gyrus	Left	19	−48	−79	13	4.48	
Middle occipital gyrus	Left	19	−36	−85	19	4.4	
Superior frontal gyrus	Right	8	30	41	43	4.51	9
Middle frontal gyrus	Right	8	42	35	37	3.61	
Middle frontal gyrus	Right	6	30	8	64	4.4	11
Pyramis	Right		6	−76	−26	4.31	88
Pyramis	Left		−6	−73	−26	4.16	
Posterior cingulate	Right	30	18	−55	13	4.16	26
Posterior cingulate	Right	30	24	−58	22	3.6	
Inferior parietal lobule	Right	40	57	−40	40	4.04	40
Insula	Right	13	33	29	4	3.8	19
Middle frontal gyrus	Right	9	39	47	25	3.7	11
Inferior frontal gyrus	Left	46	−45	44	7	3.64	5
*fear > neutral*							
Parahippocampal gyrus	Right	30	21	−43	−5	4.68	95
Insula	Right	13	36	14	−14	4.52	104
Inferior frontal gyrus	Right	45	48	23	−2	4.29	
Insula	Right	13	33	29	−2	3.82	
Culmen	Left		−18	−46	−8	4.42	90
Culmen	Right		3	−40	−2	3.84	
Culmen	Left		−18	−37	−17	3.54	
Uvula	Right		0	−67	−29	4.11	17
Thalamus (medial dorsal nucleus)	Right		6	−10	13	4.1	36
Posterior cingulate	Right	30	18	−55	13	4.02	28
Posterior cingulate	Right	31	24	−61	22	3.38	
Precuneus	Right	7	9	−49	55	3.96	27
Precuneus	Right	7	9	−52	46	3.37	
Thalamus	Left		0	−31	7	3.92	30
Cingulate gyrus	Left	31	−15	−37	43	3.82	7
Precuneus	Left	7	−9	−52	58	3.54	9
Insula	Left	13	−39	23	1	3.54	11
Supramarginal gyrus	Right	40	63	−49	31	3.53	9

Note: threshold was set at *p* < 0.001 (uncorrected). Cluster size is represented by *k*. MNI = Montreal Neurological Institute.

**Table 3:** Brain regions associated with the effects of the sadness induction.

Brain regions	Hemisphere	Brodmann's area	MNI coordinates			*t*(24)	*k*
			*x*	*y*	*z*		
*(sadness > neutral) ∩ (sadness > fear) (conjunction)*							
Superior temporal gyrus/superior temporal sulcus	Right	38	51	11	−20	7.11	127
Superior temporal gyrus/superior temporal sulcus	Right	41	51	−31	4	4.41	57
Superior temporal gyrus/superior temporal sulcus	Right	22	60	−37	10	3.41	
Superior temporal gyrus/superior temporal sulcus	Left	38	−48	11	−17	4.12	36
Superior temporal gyrus/superior temporal sulcus	Left	38	−48	8	−26	4.02	
Superior frontal gyrus	Right	9	12	53	28	3.53	17
*sadness > fear*							
Superior temporal gyrus/superior temporal sulcus	Right	38	54	11	−17	7.74	449
Superior temporal gyrus/superior temporal sulcus	Right	22	66	−10	−2	6.27	
Superior temporal gyrus/superior temporal sulcus	Right	41	54	−31	7	6.26	
Superior temporal gyrus/superior temporal sulcus	Left	22	−60	−4	1	5.3	287
Superior temporal gyrus/superior temporal sulcus	Left	38	−48	11	−17	5.02	
Middle temporal gyrus	Left	22	−51	−37	1	4.92	
Superior frontal gyrus	Right	9	15	53	25	4.66	279
Superior frontal gyrus	Right	6	15	23	52	3.72	
Medial prefrontal cortex/medial frontal gyrus	Right	32	21	20	43	3.66	
Parahippocampal gyrus	Left	27	−27	−28	−5	4.27	30
Superior frontal gyrus	Left	9	−15	50	25	4.14	143
Medial prefrontal cortex/medial frontal gyrus	Left	8	−15	38	37	3.59	
Medial prefrontal cortex/medial frontal gyrus	Left	9	−24	50	7	3.56	
Postcentral gyrus	Left	3	−51	−16	55	4.05	11
Insula	Left	13	−48	−16	22	3.77	46
Insula	Left	13	−39	−16	25	3.72	
*sadness > neutral*							
Superior temporal gyrus/superior temporal sulcus	Right	38	51	11	−20	7.11	209
Parahippocampal gyrus (amygdala)	Left		−18	−7	−14	4.84	29
Insula	Right	13	39	14	−14	4.7	
Superior temporal gyrus/superior temporal sulcus	Right	41	51	−31	4	4.41	58
Superior temporal gyrus/superior temporal sulcus	Right	22	63	−37	10	3.42	
Superior temporal gyrus/Superior temporal sulcus	Left	38	−48	11	−17	4.12	36
Superior temporal gyrus/superior temporal sulcus	Left	38	−48	8	−26	4.02	
Inferior frontal gyrus	Right	47	48	32	−8	3.73	15
Inferior frontal gyrus	Right	45	51	23	−2	3.42	
Medial prefrontal cortex/medial frontal gyrus	Right	6	9	53	31	3.63	28
Medial prefrontal cortex/medial frontal gyrus	Right	9	6	59	16	3.48	

Note: threshold was set at *p* < 0.001 (uncorrected). Cluster size is represented by *k*. MNI = Montreal Neurological Institute.

## Data Availability

The datasets generated during and/or analyzed during the current study are available from the corresponding author Jing Luo (luoj@psych.ac.cn) on reasonable request.
